# Serological risk factors for steroid-induced osteonecrosis in HIV men: a Bayesian case–control study

**DOI:** 10.3389/fmed.2025.1650001

**Published:** 2025-10-14

**Authors:** Yunxiao Ji, Kangpeng Li, Rugang Zhao, Changsong Zhao, Qiang Zhang

**Affiliations:** 1 Beijing Ditan Hospital, Capital Medical University, Beijing, China; 2 Department of Orthopedics, Peking University Third Hospital, Beijing, China

**Keywords:** HIV, hormonal necrosis of the femoral head, lipid metabolism, hypercoagulability, Bayesian regression

## Abstract

**Background:**

Human immunodeficiency virus (HIV)-infected individuals face diagnostic challenges for non-traumatic hormonal necrosis of femoral head (SONFH), as current imaging methods lack sensitivity/specificity and reliable biomarkers remain elusive. While coagulation disorders and dyslipidemia are known risk factors, evidence in HIV populations is limited.

**Methods:**

This case–control study enrolled 48 HIV-positive males with SONFH and 50 controls from Beijing Ditan Hospital (2021–2025). We analyzed demographic, coagulation, inflammatory, and metabolic markers. Random forest selected top 20 predictors, followed by Bayesian regression to assess associations (reported as OR, 95% CI, posterior probabilities, and Bayes factors).

**Results:**

Triglycerides (TG) showed the strongest SONFH association (OR = 85.911, 95% CI: 4.733–3078.857; BF = 82.048). Fibrinogen degradation products (FDP) (OR = 6.968, 95% CI: 1.485–51.347; BF = 6.692) and plasma thromboplastin antecedent (PTA) (OR = 2.890, 95% CI: 1.131–11.146; BF = 2.046) also demonstrated significant risk associations. Prolonged prothrombin time (PT) was protective (OR = 0.008, 95% CI: 0.0002–0.234; BF = 37.897).

**Conclusion:**

Elevated triglycerides, FDP, and PTA significantly increase the risk of SONFH in HIV patients, while prolonged PT may be protective. These serum markers could guide early intervention, though larger prospective studies are needed.

## 1 Introduction

Human immunodeficiency virus (HIV) infection remains a critical global health issue. According to the latest statistics from the Joint United Nations Programme on HIV/AIDS (UNAIDS), by the end of 2023, approximately 39.9 million (36.1 million–44.6 million) people worldwide were living with HIV, of whom 30.7 million were receiving antiretroviral therapy (ART) (1). While highly active antiretroviral therapy (HAART) has significantly prolonged the life expectancy in patients with AIDS (2), the chronic complications associated with long-term HIV infection have become increasingly prominent. Hormonal necrosis of the femoral head (SONFH) has been recognized as one of the important complications of long-term HIV infection. A meta-analysis by Mont et al. (3) found a 10% increase in the probability of osteonecrosis with a 10 mg per day increase in corticosteroid dose. HIV-infected individuals often require long-term or high-dose glucocorticoids for treatment of associated complications due to abnormalities in the immune system and chronic inflammatory states, placing the HIV population at increased risk of SONFH compared to the general population. The pathology is characterized by hormonal interference with bone and lipid metabolism and impairment of microcirculation and H-vessel formation, which ultimately leads to fracture of bone trabeculae (4). SONFH is a progressive disease, with approximately 80% of patients progressing to femoral head collapse within 1–3 years without effective treatment (5), severely affecting patients’ quality of life and functional activities.

Early diagnosis of HIV-associated SONFH remains challenging. Clinical diagnosis of SONFH primarily relies on imaging techniques. Although X-ray is the most commonly used initial screening tool due to its simplicity and low cost, it lacks sensitivity in the early stages of the disease, often delaying timely intervention and resulting in irreversible bone damage (6). Magnetic resonance imaging (MRI), currently regarded as the most sensitive and specific imaging modality for early SONFH diagnosis, still has limitations. The sensitivity of MRI for early-stage SONFH varies widely, sometimes dropping as low as 46%, and its detection rate for small lesions remains suboptimal. Additionally, single MRI screenings in high-risk populations (HIV-infected individuals, long-term steroid users, etc.) may yield false-positive or false-negative results (7–9). The lack of specific biomarkers for HIV-associated SONFH further complicates early risk prediction and diagnosis (10).

The difficulty in early diagnosis of HIV-associated SONFH often leads to missed intervention opportunities, ultimately necessitating joint replacement surgery, which severely impacts patients’ quality of life and increases healthcare costs. Current imaging-based diagnostic approaches suffer from insufficient sensitivity or high costs, while the absence of specific biomarkers limits screening efficiency in high-risk populations. Therefore, identifying serum marker differences holds significant clinical value for early warning and diagnosis of HIV-associated SONFH.

Recent studies have explored serum biomarkers for early diagnosis of HIV-associated SONFH. For instance, Morse et al. (11) used linear regression to compare HIV-infected patients with and without SONFH, identifying potential associations between SONFH and markers such as D-dimer and C-reactive protein (CRP). However, existing research lacks comprehensive clinical cohort studies examining the relationship between lipid metabolism indicators and SONFH in HIV-infected individuals. Moreover, most studies rely on traditional statistical methods (such as univariate analysis or multiple linear regression), which are poorly suited for high-dimensional data and small sample sizes, compromising the stability and reproducibility of biomarker screening.

To address these gaps, this study innovatively employs Bayesian regression in a case–control design, systematically analyzing coagulation function, inflammatory markers, and lipid metabolism parameters in HIV-infected individuals with and without SONFH. By establishing a multidimensional biomarker profile (encompassing lipid metabolism, coagulation, and inflammation) for HIV-associated SONFH and applying Bayesian regression to optimize small-sample data analysis, this study effectively mitigates issues of multicollinearity and overfitting, enhancing biomarker reliability. These findings not only address the limitations of existing methods but also lay the groundwork for future large-scale validation.

## 2 Methods

### 2.1 Participants

This study used a case–control study to include 48 HIV-positive male patients with SONFH who were admitted to the Department of Orthopedics of Beijing Ditan Hospital affiliated to Capital Medical University from January 2021 to January 2025 as the case group, and 50 male patients who were HIV-positive with hormones but did not develop femoral head necrosis as the control group. The diagnostic criteria for HIV infection and ONFH were based on the *Chinese Guidelines for Diagnosis and Treatment of HIV/AIDS (2021 Edition)* and the *Chinese Guidelines for Clinical Diagnosis and Treatment of Osteonecrosis of the Femoral Head in Adults (2020)* (10).

All candidates first had to fulfil three prerequisite criteria: (1) laboratory-confirmed HIV infection in males aged ≥18 years; (2) a documented history of exogenous glucocorticoid exposure; and (3) fully traceable medical records that included a detailed HIV-disease timeline, evaluable hip imaging, and a complete baseline laboratory panel. After these prerequisites were verified, participants were assigned to one of two groups. For the case group, simultaneous fulfilment of the following was required: (1) presence of hip or groin pain, with or without concomitant knee pain or limited internal rotation; (2) definite osteonecrosis of the femoral head on radiographs, MRI, or CT; and (3) no hip trauma within the preceding 12 months and exclusion of secondary osteonecrosis attributable to ankylosing spondylitis, rheumatoid arthritis, skeletal metastases, septic arthritis of the hip, or any other confounding disorder. Conversely, the control group was defined by the absence of osteonecrosis on the same imaging modalities. Participants were excluded if any of the following applied: (1) ≥5% of critical clinical or imaging data were missing; (2) severe dysfunction of vital organs or active malignancy was present; or (3) there was a history of chronic excessive alcohol consumption.

### 2.2 Clinical data

This study is a retrospective, single-center observational study that screened patients who met the criteria by searching the in-hospital electronic medical record management system of Beijing Ditan Hospital affiliated to Capital Medical University (2021–2025). Based on the preliminary analysis of our electronic medical record system, more than 90% of patients with HIV and hormonal femoral head necrosis were male, consistent with the previously reported gender distribution of HIV-related osteonecrosis, so only male patients were included in this study to control for gender confounding factors (12). Men account for a large proportion of patients with AIDS and necrosis of the femoral head, so only male patients were included in the gender inclusion of this study. The research team extracted the following data from all the selected patients in the electronic medical record system: (1) demographic characteristics: age, marital status, smoking history, alcohol history; (2) Comorbidities: hypertension, diabetes, hepatitis B, hepatitis C, syphilis (3) HIV treatment: HAART treatment regimen, viral load, CD4^+^ T cell count, CD8^+^ T cell count, CD4^+^ T/CD8^+^ T; (4) Coagulation and inflammation indicators: prothrombin time (PT), prothrombin activity (PTA), activated partial thromboplastin time (APTT), fibrinogen (Fb), international normalized ratio (INR), D-dimer, thrombin time (TT), C-reactive protein (CRP), erythrocyte sedimentation rate (ESR) (5) Hematological indicators: complete blood count (white blood cells, neutrophils, lymphocytes, monocytes, red blood cells, hemoglobin, platelets), platelet average volume (MPV), platelet (PCT), large platelet ratio; (6) Metabolic and biochemical indicators: total cholesterol, triglycerides, high-density lipoprotein cholesterol (HDL-C), low-density lipoprotein cholesterol (LDL-C), apolipoprotein A1, apolipoprotein B, lipoprotein a (Lpa), serum calcium, blood magnesium, blood phosphorus, serum creatinine, uric acid, blood glucose, glomerular filtration rate (eGFR), alanine aminotransferase (ALT), aspartate aminotransferase (AST), albumin, globulin, white globulin ratio, cholinesterase.

### 2.3 Ethical approval and informed consent

This study strictly adhered to the principles of the *Declaration of Helsinki* and was approved by the Ethics Committee of Beijing Ditan Hospital, Capital Medical University (Approval No. DTEC-KY2023-022-02). Written informed consent was obtained from all participants. All data were anonymized and used solely for research purposes. No identifiable private information will be disclosed in any publication arising from this study.

### 2.4 Statistical analysis

R software (v4.3.3) was used for analysis. The random forest algorithm (randomForest package) screened key variables, and Spearman’s rank correlation excluded highly correlated variables. Bayesian generalized linear models estimated ORs, 95% CIs, posterior probabilities, and BFs. Markov Chain Monte Carlo (MCMC) sampling (four chains, 4,000 iterations) ensured model convergence (Rhat <1.01, ESS ≥5,000).

## 3 Results

### 3.1 Demographic characteristics

A total of 98 HIV-positive patients were included in this study, of which 50 (51%) did not develop necrosis of the femoral head (control group) and 48 (49%) were diagnosed with necrosis of the femoral head (case group), all of whom were male. The demographic characteristics, living habits, underlying diseases, and HAART treatment regimens were compared between the two groups, and the results were as follows: there was no significant difference in age distribution between the case group and the control group (*p* = 0.8), and there was no significant difference in marital status between the two groups (*p* = 0.2). In addition, in terms of lifestyle habits, 4.0% smokers in the control group and 13% smokers in the case group (*p* = 0.2), all participants had no history of alcohol consumption. In terms of comorbidities, there was no significant difference in the prevalence of hypertension, diabetes mellitus, hepatitis B, hepatitis C and syphilis between the case group and the control group (*p* > 0.05). In terms of antiviral therapy, 86% of the participants received the 3TC + TDF + EFV regimen, and there was no significant difference between the two groups (*p* = 0.10), but the proportion of viral load >40 copies/mL in the case group was significantly higher than that in the control group (23% vs. 8.0%, *p* = 0.040) (as shown in Table 1).

**Table 1:** Baseline characteristics of HIV patients stratified by osteonecrosis of the femoral head (ONFH) status.

Variables	Osteonecrosis of the femoral head			*p*-value^2^
	Overall *N* = 98^1^	0 *N* = 50^1^	1 *N* = 48^1^	
Age *n* (%)				0.8
<40	40 (41%)	21 (42%)	19 (40%)	
40–59	46 (47%)	24 (48%)	22 (46%)	
≥60	12 (12%)	5 (10%)	7 (15%)	
Femoral head necrosis staging *n* (%)				**<0.001**
None	50 (51%)	50 (100%)	0 (0%)	
Phase II	4 (4.1%)	0 (0%)	4 (8.3%)	
Phase IIIA	8 (8.2%)	0 (0%)	8 (17%)	
Phase IIIB	10 (10%)	0 (0%)	10 (21%)	
Phase IV	26 (27%)	0 (0%)	26 (54%)	
Ethnicity *n* (%)				0.4
Han	91 (93%)	45 (90%)	46 (96%)	
Hui	2 (2.0%)	1 (2.0%)	1 (2.1%)	
Manchu	3 (3.1%)	3 (6.0%)	0 (0%)	
Mongol	2 (2.0%)	1 (2.0%)	1 (2.1%)	
Marital status *n* (%)				0.2
Single	40 (41%)	24 (48%)	16 (33%)	
Married	50 (51%)	24 (48%)	26 (54%)	
Divorced	8 (8.2%)	2 (4.0%)	6 (13%)	
Smoke *n* (%)				0.2
No	90 (92%)	48 (96%)	42 (88%)	
Yes	8 (8.2%)	2 (4.0%)	6 (13%)	
Alcohol *n* (%)				
No	98 (100%)	50 (100%)	48 (100%)	
Yes	0 (0%)	0 (0%)	0 (0%)	
Hypertension *n* (%)				0.6
No	72 (73%)	38 (76%)	34 (71%)	
Yes	26 (27%)	12 (24%)	14 (29%)	
Diabetes *n* (%)				0.7
No	76 (78%)	38 (76%)	38 (79%)	
Yes	22 (22%)	12 (24%)	10 (21%)	
Hepatitis B *n* (%)				0.3
No	91 (93%)	48 (96%)	43 (90%)	
Yes	7 (7.1%)	2 (4.0%)	5 (10%)	
Hepatitis C *n* (%)				>0.9
No	96 (98%)	49 (98%)	47 (98%)	
Yes	2 (2.0%)	1 (2.0%)	1 (2.1%)	
Syphilis *n* (%)				0.6
No	68 (69%)	36 (72%)	32 (67%)	
Yes	30 (31%)	14 (28%)	16 (33%)	
Highly active antiretroviral therapy *n* (%)				
3TC + TDF + EFV				0.10
No	14 (14%)	10 (20%)	4 (8.3%)	
Yes	84 (86%)	40 (80%)	44 (92%)	
Bictegravir sodium				0.2
No	83 (85%)	40 (80%)	43 (90%)	
Yes	15 (15%)	10 (20%)	5 (10%)	
Viral load *n* (%)				**0.040**
≤40	83 (85%)	46 (92%)	37 (77%)	
>40	15 (15%)	4 (8.0%)	11 (23%)	
CD_8_^+^ T cells (cells/μL)	897 (495)	830 (340)	967 (612)	0.2
CD_4_^+^ T cells (cells/μL)	570 (401)	586 (306)	553 (483)	0.089
CD_8_^+^/CD_4_^+^ ratio	0.72 (0.50)	0.77 (0.41)	0.67 (0.58)	**0.026**
Erythrocyte sedimentation rate (mm/h)	19 (23)	11 (11)	27 (29)	**0.002**
C-Reactive protein (mg/L)	15 (32)	6 (8)	25 (43)	**<0.001**
Prothrombin time (s)	10.44 (1.50)	11.43 (1.21)	9.40 (0.99)	**<0.001**
Prothrombin time activity (%)	104 (14)	98 (13)	109 (12)	**<0.001**
Activated partial thromboplastin time (s)	33.2 (20.6)	31.0 (3.9)	35.6 (29.1)	0.6
Fibrinogen (mg/dL)	308 (93)	282 (80)	335 (99)	**0.009**
International normalized ratio	0.95 (0.51)	1.03 (0.12)	0.88 (0.72)	**<0.001**
Fibrinogen degradation products (μg/mL)	4.3 (5.6)	3.0 (6.7)	5.7 (3.7)	**<0.001**
D-dimer (μg/mL)	0.99 (1.12)	0.77 (1.14)	1.23 (1.05)	**<0.001**
Thrombin time (s)	13.90 (1.76)	14.33 (1.50)	13.46 (1.92)	**0.014**
White blood cells count (×10^9^/L)	6.89 (2.61)	6.64 (2.36)	7.15 (2.86)	0.4
Neutrophil count (×10^9^/L)	4.21 (2.31)	3.97 (1.92)	4.46 (2.66)	0.5
Lymphocyte count (×10^9^/L)	2.11 (0.97)	2.07 (0.83)	2.14 (1.11)	0.9
Monocyte count (×10^9^/L)	0.44 (0.19)	0.42 (0.18)	0.47 (0.21)	0.4
Red blood cells count (×10^12^/L)	4.48 (0.73)	4.55 (0.65)	4.40 (0.80)	0.4
Hemoglobin (g/L)	145 (19)	147 (17)	143 (20)	0.2
Platelet count (×10^9^/L)	233 (75)	213 (54)	253 (89)	**0.023**
Mean platelet volume (fL)	10.06 (0.82)	10.06 (0.86)	10.06 (0.77)	0.8
Plateletcrit (%)	0.30 (0.14)	0.22 (0.05)	0.39 (0.14)	**<0.001**
Large platelet ratio (%)	31 (12)	25 (7)	37 (13)	**<0.001**
Platelet distribution width (%)	13.5 (3.8)	11.2 (1.7)	15.9 (3.9)	**<0.001**
Total cholesterol (mmol/L)	5.08 (1.26)	4.46 (0.92)	5.73 (1.25)	**<0.001**
Triglycerides (mmol/L)	2.52 (1.81)	1.58 (1.07)	3.50 (1.92)	**<0.001**
High-density lipoprotein (mmol/L)	0.92 (0.39)	1.16 (0.32)	0.67 (0.27)	**<0.001**
Low-density lipoprotein (mmol/L)	2.93 (1.18)	2.39 (0.74)	3.49 (1.30)	**<0.001**
Apolipoprotein A1 (g/L)	1.21 (0.38)	1.47 (0.30)	0.93 (0.23)	**<0.001**
Apolipoprotein B (g/L)	1.08 (0.47)	0.73 (0.24)	1.44 (0.36)	**<0.001**
Lipoprotein(a) (mg/L)	130 (163)	16 (19)	248 (163)	**<0.001**
Calcium (mmol/L)	2.29 (0.19)	2.27 (0.11)	2.30 (0.24)	0.6
Magnesium (mmol/L)	0.90 (0.08)	0.92 (0.07)	0.89 (0.09)	0.11
Phosphate (mmol/L)	1.01 (0.21)	1.02 (0.19)	1.00 (0.23)	0.6
Creatinine (μmol/L)	75 (18)	78 (16)	73 (21)	0.3
Uric acid (μmol/L)	376 (112)	379 (116)	372 (110)	0.8
Glucose (mmol/L)	5.93 (1.33)	5.98 (1.36)	5.88 (1.32)	0.5
Estimated glomerular filtration rate (mL/min/1.73m^2^)	107 (21)	104 (17)	109 (25)	0.3
Alanine aminotransferase (U/L)	30 (20)	30 (19)	30 (22)	0.7
Aspartate aminotransferase (U/L)	23 (9)	25 (9)	21 (8)	**0.019**
Albumin (g/L)	43.3 (5.4)	43.5 (5.2)	43.1 (5.7)	0.8
Globulin (g/L)	30.4 (4.8)	30.5 (4.9)	30.3 (4.7)	0.7
Albumin-to-globulin ratio	1.45 (0.25)	1.46 (0.26)	1.44 (0.24)	0.8
Cholinesterase (U/L)	8,064 (2,737)	8,883 (2,116)	7,210 (3,056)	**0.002**

^1^Data are presented as mean ± standard deviation (SD) for continuous variables and *n* (%) for categorical variables. ^2^Group comparisons were performed using Wilcoxon rank-sum test (non-normal continuous data), Fisher’s exact test (categorical data with expected counts <5), or Pearson’s chi-square test (categorical data with expected counts ≥5). Significant differences (*p* < 0.05) are highlighted in bold.

HAART, highly active antiretroviral therapy; CD_8_^+^ T cells, cluster of differentiation 8-positive T cells; CD_4_^+^ T cells, cluster of differentiation 4-positive T cells; ESR, erythrocyte sedimentation rate; CRP, C-reactive protein; PT, prothrombin time; PTA, prothrombin activity; APTT, activated partial thromboplastin time; Fb, fibrinogen; FDP, fibrin degradation products; INR, international normalized ratio; TT, thrombin time; WBC, white blood cell count; RBC, red blood cell count; HGB, hemoglobin; PLT, platelet count; MPV, mean platelet volume; PCT, plateletcrit; P-LCR, platelet large cell ratio; PDW, platelet distribution width; TC, total cholesterol; TG, triglycerides; HDL-C, high-density lipoprotein cholesterol; LDL-C, low-density lipoprotein cholesterol; ApoA1, apolipoprotein A1; ApoB, apolipoprotein B; Lp(a), lipoprotein(a); Ca, serum calcium; Mg, serum magnesium; P, serum phosphate; Scr, serum creatinine; UA, uric acid; GLU, plasma glucose; GFR, estimated glomerular filtration rate; ALT, alanine aminotransferase; AST, aspartate aminotransferase; ALB, albumin; GLB, globulin; A/G, albumin/globulin ratio; CHE, cholinesterase.

### 3.2 Random forest-based predictor screening

This study employed the random forest algorithm to evaluate variable importance and perform feature selection for predicting osteonecrosis of the femoral head (ONFH), aiming to identify predictors significantly associated with the condition. Initially, the randomForest package in R was used to construct a preliminary model with ONFH as a binary outcome variable, incorporating all available predictors. To ensure reproducibility, a random seed was pre-set (set.seed = 123). By analyzing the out-of-bag (OOB) error curve as a function of the number of decision trees, the mtry parameter (number of variables considered at each node split) was set to 8, approximately equal to the square root of the total number of variables—a common practice for classification problems.

Variable importance was assessed using two metrics: the mean decrease in Gini index (MeanDecreaseGini) and the mean decrease in accuracy (MeanDecreaseAccuracy). MeanDecreaseGini evaluates the discriminative power of each variable by measuring its average contribution to reducing impurity during node splits; a higher value indicates greater importance in distinguishing between classes. MeanDecreaseAccuracy assesses predictive importance by observing the increase in OOB error after random permutation of the variable; a significant decrease in model accuracy upon permutation suggests a substantial contribution of the variable to prediction. This study primarily ranked all predictors in descending order based on MeanDecreaseGini scores, as this metric more stably reflects discriminative ability in classification tasks. The top 19 variables with the highest importance scores under this criterion were selected for subsequent analysis, including: *apolipoprotein B, apolipoprotein A1, prothrombin time, plateletcrit, lipoprotein(a), triglycerides, high-density lipoprotein, platelet distribution width, international normalized ratio, large platelet ratio, total cholesterol, low-density lipoprotein, cholinesterase, fibrin degradation products, prothrombin activity, CD8^+^ T-cell count, C-reactive protein, estimated glomerular filtration rate, and aspartate aminotransferase*.

Model performance was validated using the OOB error rate, with the final model achieving an error rate of 1.02%. To address potential multicollinearity among the selected variables, variance inflation factor (VIF) testing (threshold set at 5) will be subsequently performed. Variables passing the test (VIF <5) will be included in a Bayesian regression model for further analysis.

### 3.3 Bayesian regression model construction

**Figure 1:**
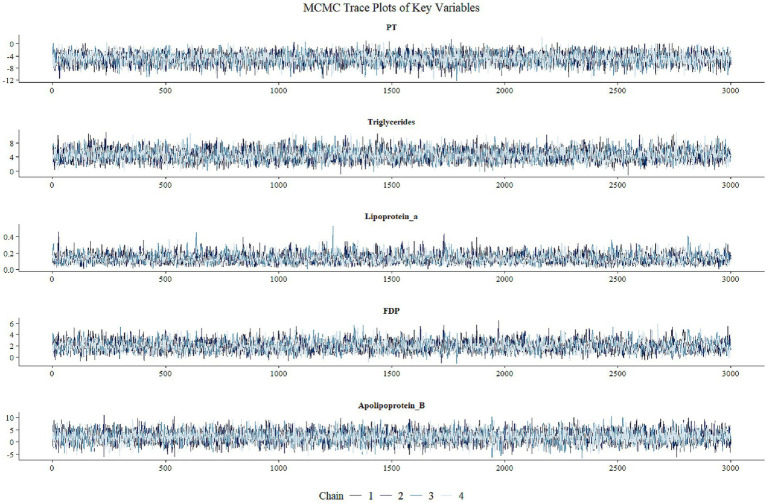
Trace plots of key parameters in Markov Chain Monte Carlo (MCMC) sampling. The trace plots demonstrate sampling trajectories for five parameters: prothrombin time (PT), triglycerides, lipoprotein(a), fibrinogen degradation products (FDP), and apolipoprotein B. Four independent chains (Chain 1–4) are represented by distinct colors/line types, with the *x*-axis indicating iteration number and the *y*-axis showing parameter values. The overlapping and stable fluctuations of chains during the warm-up period suggest successful convergence.

**Figure 2:**
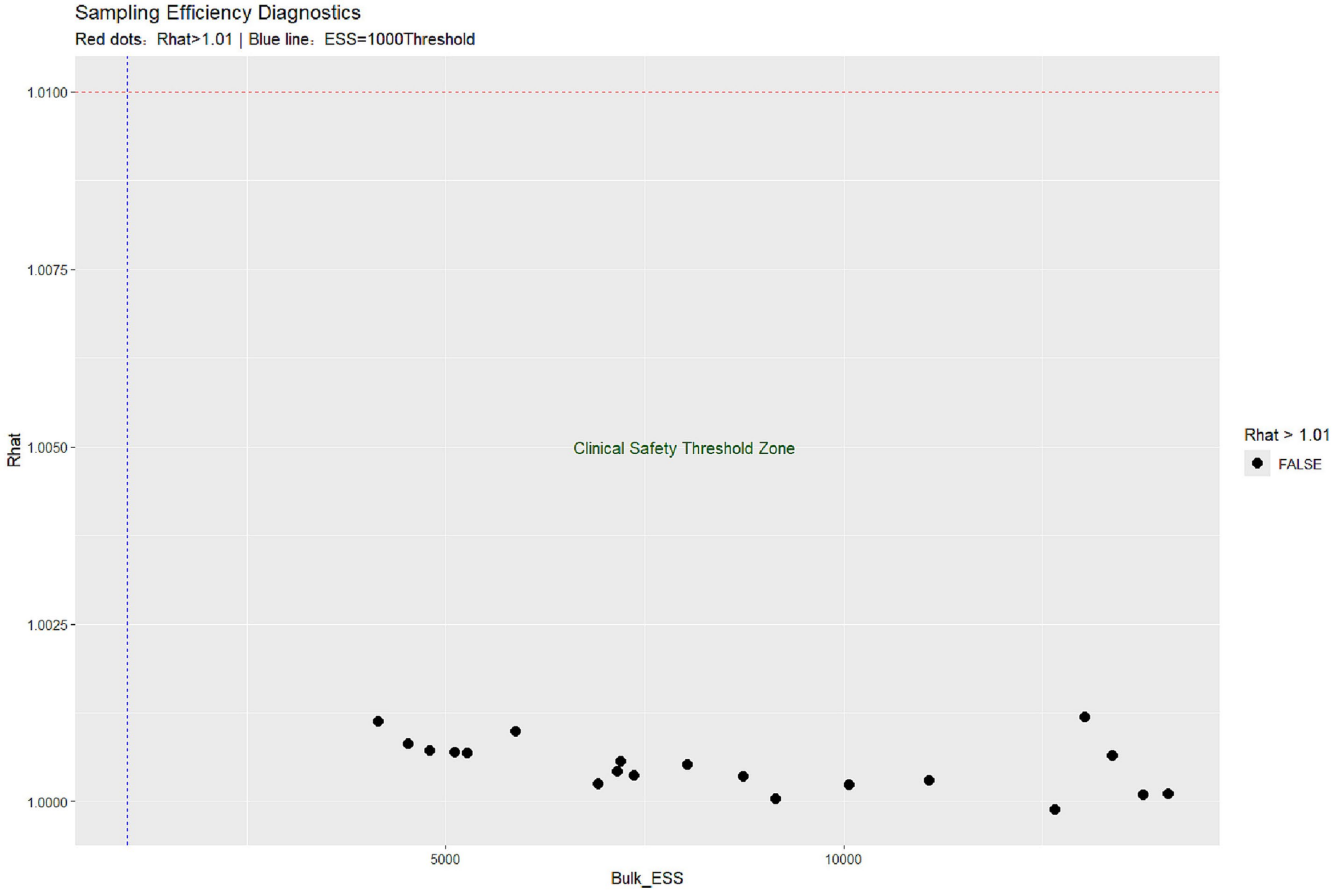
Convergence diagnostics for Bayesian Markov Chain Monte Carlo (MCMC) sampling. (a) R-hat values for all parameters (target <1.01, optimal ≈1.0). Red dots would indicate potentially non-convergent parameters (R-hat >1.01), though none were observed in this analysis (maximum R-hat = 1.0005). (b) Effective sample size (ESS) distribution with clinical safety threshold (blue line at ESS = 1,000). All parameters exceeded the conservative threshold (minimum bulk ESS = 5,000), indicating excellent posterior sampling efficiency. Absence of red dots confirms convergence was achieved across all four independent chains (4,000 iterations per chain, including 1,000 warm-up).

**Figure 3:**
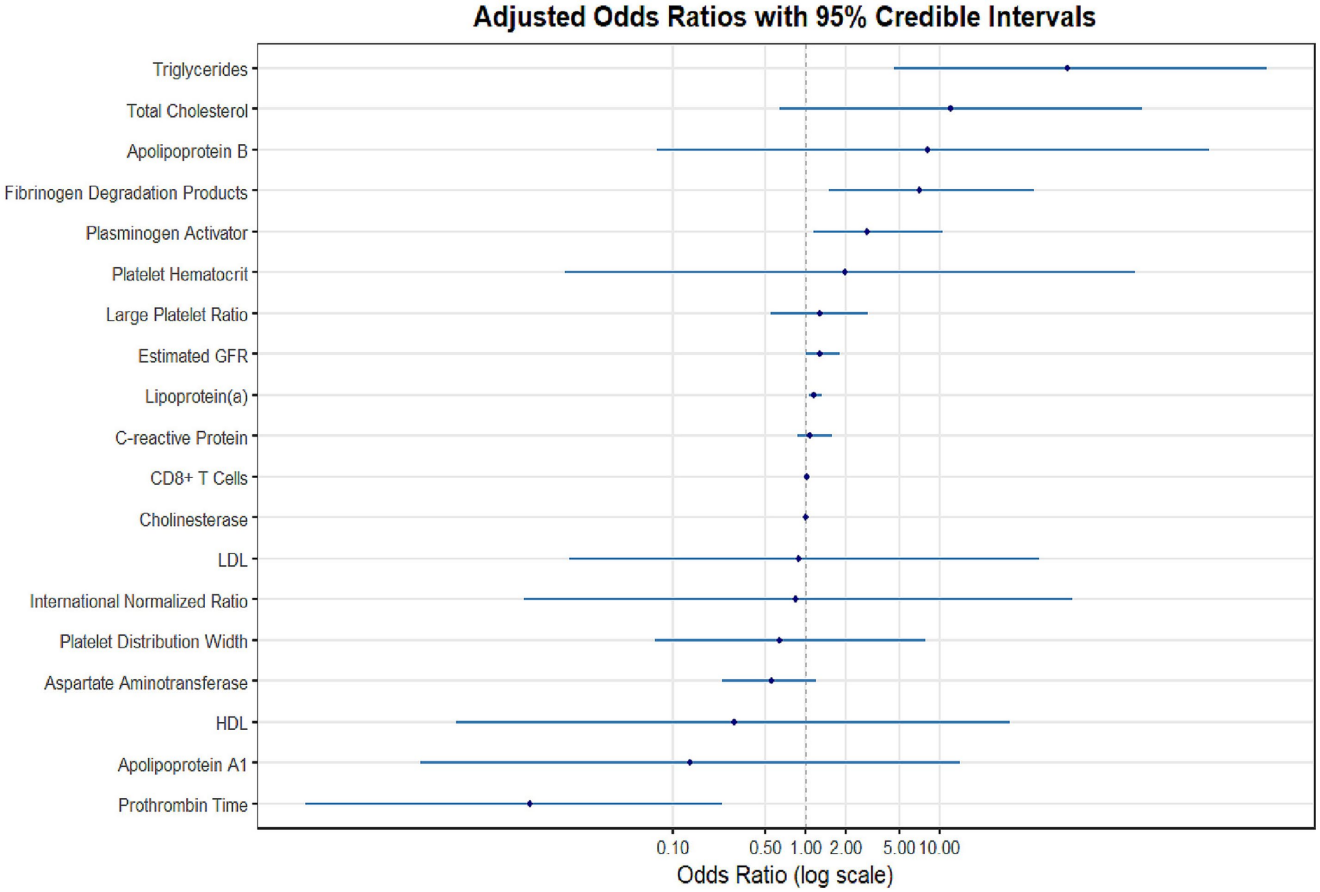
Forest plot of adjusted odds ratios (ORs) with 95% credible intervals for NONFH risk factors in HIV patients. Bayesian regression analysis results are presented on a logarithmic scale. Key findings include: triglycerides (OR = 85.91, 95% CI: 4.73–3078.86) and fibrinogen degradation products (OR = 6.97, 95% CI: 1.49–51.35) demonstrated the strongest risk effects, while prothrombin time showed significant protective effects (OR = 0.008, 95% CI: 0.0002–0.23). The vertical dashed line (OR = 1) indicates the null value.

In this study, Bayesian regression model was used to analyze the association between variables and target outcomes. During model construction, all continuous variables were assigned normal prior distributions (mean = 0, standard deviation = 10), while categorical variables were assigned uniform priors. This prior specification follows the principle of weakly informative priors, aiming to reduce subjective bias while allowing the data to dominate the posterior inference. The model uses the Markov Chain Monte Carlo (MCMC) method for parameter estimation, and sets up four independent MCMC chains, each chain runs 4,000 iterations (including 1,000 warm-up periods), and retains 3,000 valid samples. The parameter space of each chain in the trajectory diagram (Figure 1) is fully explored and evenly mixed, indicating that the model has good convergence. The convergence diagnostics (Figure 2) showed that there were no anomalies in Rhat values above 1.01 for all parameters, indicating good interchain convergence. In addition, the effective sample size (ESS) was much higher than the clinical safety threshold (Bulk_ESS ≥5,000), indicating that the reliability of posterior distribution sampling met the statistical requirements. The results of the analysis of the effects of key variables are shown in Table 2 and Figure 3. Prothrombin time (PT) showed a significant protective effect (median OR = 0.008, 95% CI: 0.0002–0.234), a posteriori probability of 0.002, and a Bayesian factor (BF) of 37.897, supporting it as a strong protective factor. Both triglyceride (TG) and fibrinogen degradation products (FDP) were identified as strong risk factors. Specifically, TG exhibited an exceptionally high odds ratio (OR) of 85.911 (95% CI: 4.733–3078.857), with a posterior probability of 0.999 and a Bayes factor (BF) of 82.048. Meanwhile, FDP showed an OR of 6.968 (95% CI: 1.485–51.347), accompanied by a posterior probability of 0.994 and a BF of 6.692. Both the two variables demonstrated strong statistical evidence. It is noteworthy that triglyceride (TG) exhibited an exceptionally wide confidence interval, indicating substantial uncertainty in its estimation. This may be attributed to limited sample size or potential influence from extreme values, warranting cautious interpretation of the results. Lipoprotein a, although small (OR = 1.139), had a 95% CI of 1 (1.050–1.302) and a posteriori probability of 1 and BF = 50.467, suggesting that it was a mild but stable risk factor. The OR confidence intervals for the remaining variables ranged from 1 to 1, and were not statistically significant.

**Table 2:** Adjusted odds ratios, posterior probabilities and Bayesian evidence for key predictors.

Variable	Median OR (95% CI)	Posterior probability	BF	Clinical interpretation
Apolipoprotein B	8.394 (0.084, 992.043)			
Apolipoprotein A1	0.149 (0.001, 15.041)			
Prothrombin time (PT)	0.008 (0.0002, 0.234)	0.002	37.897	Significant protective factor
Platelet hematocrit (PCT)	2.002 (0.167, 291.720)			
Lipoprotein a	1.139 (1.050, 1.302)	1	50.467	Mild but well-supported risk factor
Triglycerides (TG)	85.911 (4.733, 3078.857)	0.999	82.048	Strong risk factor
High-density lipoprotein (HDL)	0.272 (0.002, 33.397)			
Mean platelet volume distribution width (PDW)	0.619 (0.071, 7.770)			
International normalized ratio (INR)	0.859 (0.008, 96.273)			
Large platelet ratio (P-LCR)	1.275 (0.522, 2.895)			
Total cholesterol (TC)	11.978 (0.622, 336.412)			
Low-density lipoprotein (LDL)	0.854 (0.016, 59.605)			
Cholinesterase	0.998 (0.995, 1.001)			
Fibrinogen degradation products (FDP)	6.968 (1.485, 51.347)	0.994	6.692	Decisive risk factor
Plasma thromboplastin antecedent (PTA)	2.890 (1.131, 11.146)	0.989	2.046	Strong risk factors
CD_8_^+^ T cells	1.006 (0.991, 1.023)			
C-Reactive protein (CRP)	1.066 (0.855, 1.585)			
Estimated glomerular filtration rate (EGFR)	1.272 (0.992, 1.780)			
Aspartate aminotransferase (AST)	0.551 (0.229, 1.823)			

Adjusted odds ratios (OR) with 95% credible intervals (CI) are presented for key predictors, along with posterior probabilities and Bayes factors (BF). OR >1 indicates increased risk, while OR <1 suggests protective effects. BF interpretation follows conventional thresholds: 1–3 (weak evidence), 3–10 (moderate evidence), 10–30 (strong evidence), 30–100 (very strong evidence), and >100 (decisive evidence). Posterior probabilities represent the likelihood of non-null associations. Final clinical interpretations integrate OR magnitude, BF strength, and posterior probability thresholds.

## 4 Discussion

Nontraumatic femoral head necrosis is a pathological state in which blood flow to the femoral head is interrupted by a variety of complex nontraumatic factors (corticosteroid use, excessive alcohol consumption, metabolic disorders, coagulation abnormalities, etc.), resulting in persistent hypoxia and nutritional deficiencies in the bone (13, 14). Among them, hormonal femoral head necrosis is the most common clinical subtype, and its pathological mechanism involves microvascular injury, bone metabolism imbalance and H-type vascular dysfunction (4). The rate is significantly higher than that of the general population, which is about 100 times higher than that of the general population (15). As a common subtype of non-traumatic femoral head necrosis, SONFH patients also lack specific clinical symptoms in the early stage of the disease, and it is difficult to diagnose, and most patients who seek medical treatment for pain often have progressed to stage III and IV (ARCO stage), Ohzono et al. (16) found that patients with HIV and stage III hormonal femoral head necrosis had femoral head necrosis and collapse within an average of 11 months (12). Therefore, it is of great clinical significance to explore the application value of meaningful serological indicators in the early diagnosis of SONFH in HIV patients to slow down the disease progression of femoral head necrosis in HIV patients and improve the quality of life.

Hypercoagulability and abnormal lipid metabolism have been found to be independent risk factors for SONFH (17, 18). However, the current research based on the special population of AIDS is still insufficient, and this study explored the association between coagulation function and lipid metabolism indexes and SONFH in AIDS patients through Bayesian regression, which provides new evidence support for the clinical management of this high-risk group. This study found that lipid dystrophy was particularly prominent in patients with AIDS and SONFH. Notably, triglyceride (TG) exhibited an exceptionally high adjusted odds ratio (OR) of 85.911 (95% CI: 4.733–3078.857), with a posterior probability of 0.999 and a Bayes factor (BF) of 82.048, statistically suggesting that TG is likely a strong risk factor for steroid-induced osteonecrosis of the femoral head (SONFH). However, it is important to note that the exceptionally wide 95% confidence interval reflects substantial uncertainty in this estimate under the current sample size, which may be attributable to limited statistical power, potential extreme values, or model instability. Therefore, despite the strong evidence indicated by the Bayesian measures, the magnitude of the association between TG and SONFH should be interpreted with caution. This finding is consistent with previous reports by Kuroda et al. (19) in a systemic lupus erythematosus cohort and Yu et al. (20) in a general population, supporting the pathological mechanism whereby elevated TG levels may increase osteonecrosis risk by promoting fat embolism and microvascular obstruction. Nevertheless, given the high degree of uncertainty in the effect size estimation for TG in our study, the strength of this association warrants further validation in large-scale prospective studies. We propose that the current study preliminarily indicates a significant positive correlation between TG and SONFH in people with HIV; however, the precise effect size and broader clinical relevance remain to be confirmed in future research.

On the other hand, hypercoagulability-related indicators also showed a significant association with SONFH in the AIDS population. The OR of fibrinogen degradation products (FDP) was 6.968 (95% CI: 1.485–51.347, BF = 6.692) and the OR of plasma thromboplastin precursor (PTA) was 2.890 (95% CI: 1.131–11.146, BF = 2.046), indicating that coagulation abnormalities may increase the risk of SONFH in AIDS populations. Studies have shown that imbalance in the coagulation-fibrinolytic system may be more pronounced in patients with AIDS due to long-term immunosuppression and chronic inflammation (21). In contrast, the OR of prothrombin time (PT) was 0.008 (95% CI: 0.0002–0.234, BF = 37.897), suggesting that prolonged PT may have a protective effect, consistent with the potential value of anticoagulation in the prevention of SONFH (22).

In this study, Bayesian statistical methods were used to analyze the risk factors of SONFH, which effectively solved the instability of the results of small samples of traditional statistical methods, and the researchers introduced reasonable prior information in Bayesian regression to ensure reliable statistical inference in the case of limited samples, which is particularly important for the study of special populations such as AIDS complicated with SONFH, which is relatively rare in clinical practice. In addition, in small-sample studies, the uncertainty of parameter estimation is more fully reflected through the posterior probability distribution and Bayesian factor.

However, this study has several limitations. First, the results of the Bayesian analysis are partially dependent on the specification of prior distributions. Although weakly informative priors were used to minimize subjective bias, the choice of priors may still potentially influence posterior inference, particularly given the limited sample size. This issue is especially evident in the estimation of variables such as triglyceride (TG), whose extremely wide 95% confidence interval (4.733–3078.857) not only reflects insufficient estimation precision under the current sample but also suggests that the results may be sensitive to prior specification. Therefore, the key findings of this study should be validated in future larger-sample studies using different prior settings in sensitivity analyses to verify the robustness of the conclusions. Second, due to the retrospective nature of the study design, the cumulative dosage of glucocorticoids could not be accurately obtained, which significantly limited the assessment of dose–response relationships. The absence of this critical information may introduce bias into the risk evaluation of glucocorticoid-induced osteonecrosis of the femoral head (GIONFH), thereby affecting a comprehensive understanding of the disease mechanism. Furthermore, although baseline analyses showed no significant differences between the case and control groups in most demographic characteristics, comorbidities, and antiretroviral therapy regimens, and adjustments were made for relevant variables, residual confounding factors cannot be completely ruled out in a retrospective study. It is noteworthy that 13% of the patients in the case group had a history of smoking, compared to only 4.0% in the control group (*p* = 0.2). Although the difference was not statistically significant, smoking—a known risk factor influencing lipid metabolism and microcirculation—may still introduce non-negligible confounding effects. Additionally, other unmeasured potential confounding variables, such as body mass index (BMI), physical activity, nutritional status, and detailed alcohol consumption, may indirectly contribute to non-traumatic osteonecrosis of the femoral head (NONFH) by affecting bone metabolism and vascular health. Moreover, although differences such as viral load (a significantly higher proportion of cases had >40 copies/mL) were adjusted for in the analysis, residual effects and interactions with other factors may still influence the conclusions. Future studies should prioritize prospective cohort designs, standardize the collection of glucocorticoid usage records and cumulative doses, and systematically incorporate lifestyle indicators—including BMI, smoking intensity, alcohol use, and physical activity—as well as more detailed virological and immunological parameters. By expanding the sample size and enhancing the comprehensiveness of covariate control, it will be possible to more accurately identify independent risk factors and underlying mechanisms for NONFH in people with HIV.

## 5 Conclusion

In summary, this study preliminarily suggests that among people with HIV, dyslipidemia—as indicated by elevated serum triglyceride (TG) levels—and a hypercoagulable state, reflected by increased fibrinogen degradation products (FDP) and prothrombin activity (PTA), may be associated with a higher risk of non-traumatic osteonecrosis of the femoral head (NONFH). Meanwhile, prolonged prothrombin time (PT) appears to be correlated with a lower risk of NONFH. These findings highlight the importance of regular monitoring and evaluation of lipid metabolism and coagulation function in the clinical management of people with HIV. In particular, for individuals who have developed dyslipidemia or coagulation abnormalities, enhanced early screening and follow-up for NONFH should be considered. Additionally, the potential preventive value of lipid-lowering and anticoagulant therapies in reducing the risk of osteonecrosis warrants careful assessment. It should be noted, however, that the effect estimates of certain indicators—such as TG, which had an exceptionally wide confidence interval (95% CI: 4.733–3078.857)—exhibit considerable uncertainty. Therefore, these conclusions require further validation through large-scale prospective studies.

## Data Availability

The raw data supporting the conclusions of this article will be made available by the authors, without undue reservation.
