# Correction: Serological risk factors for steroid-induced osteonecrosis in HIV men: a Bayesian case–control study

**DOI:** 10.3389/fmed.2025.1734604

**Published:** 2025-11-10

**Authors:** Yunxiao Ji, Kangpeng Li, Rugang Zhao, Changsong Zhao, Qiang Zhang

**Affiliations:** 1 Beijing Ditan Hospital, Capital Medical University, Beijing, China; 2 Department of Orthopedics, Peking University Third Hospital, Beijing, China

**Keywords:** HIV, hormonal necrosis of the femoral head, lipid metabolism, hypercoagulability, Bayesian regression

**Error in author list**

The order of authors in the author list of the published paper was erroneously given as:

Yunxiao Ji^1^, Kangpeng Li^2^, Rugang Zhao^1*^, Changsong Zhao^1^ and Qiang Zhang^1*^

^1^Beijing Ditan Hospital, Capital Medical University, Beijing, China

^2^Department of Orthopedics, Peking University Third Hospital, Beijing, China

^*^CORRESPONDENCE

Qiang Zhang, Rugang Zhao

**The correct author list reads:**

Yunxiao Ji^1†^, Kangpeng Li^2†^, Rugang Zhao^1*^, Changsong Zhao^1^ and Qiang Zhang^1*^

^†^These authors have contributed equally to this work

^1^Beijing Ditan Hospital, Capital Medical University, Beijing, China

^2^Department of Orthopedics, Peking University Third Hospital, Beijing, China

^*^CORRESPONDENCE

Qiang Zhang, Rugang Zhao

The original version of this article has been updated.

